# Supplementary material for: Roles of CgEde1 and CgMca in Development and Virulence of Colletotrichum gloeosporioides
Source: Int J Mol Sci. 2024 Mar 3;25(5):2943. doi: 10.3390/ijms25052943 (PMC10932086; doi:10.3390/ijms25052943)
Supplement: Supplementary file 1 [file ijms-25-02943-s001.zip › ijms-2882709-supplementary.pdf]

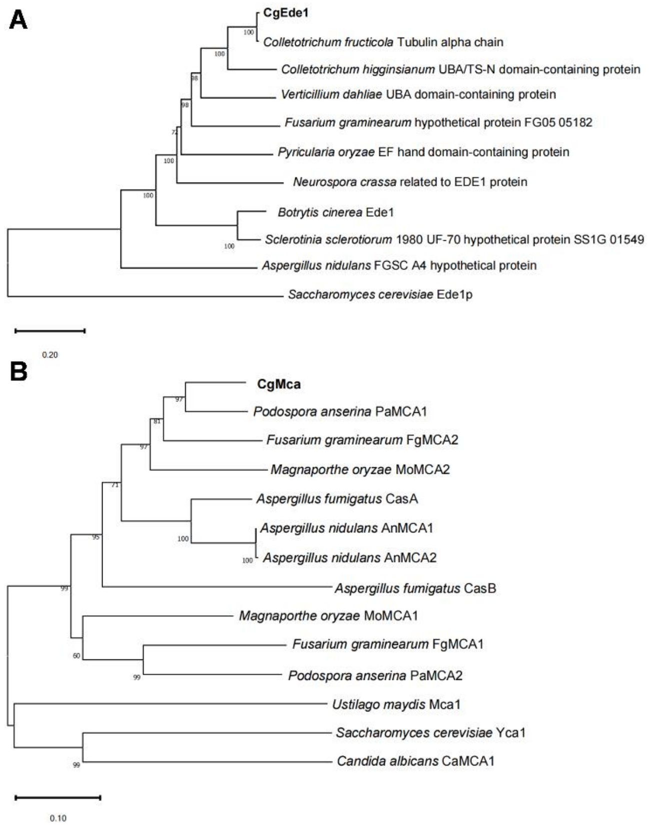

Figure S1 Phylogenetic analysis of CgEde1 and CgMca. (**A**) The phylogenetic relationship of the CgEde1 and Ede1 proteins from some other fungi. (**B**) The phylogenetic relationship of the CgMca and metacaspase proteins from some other fungi.

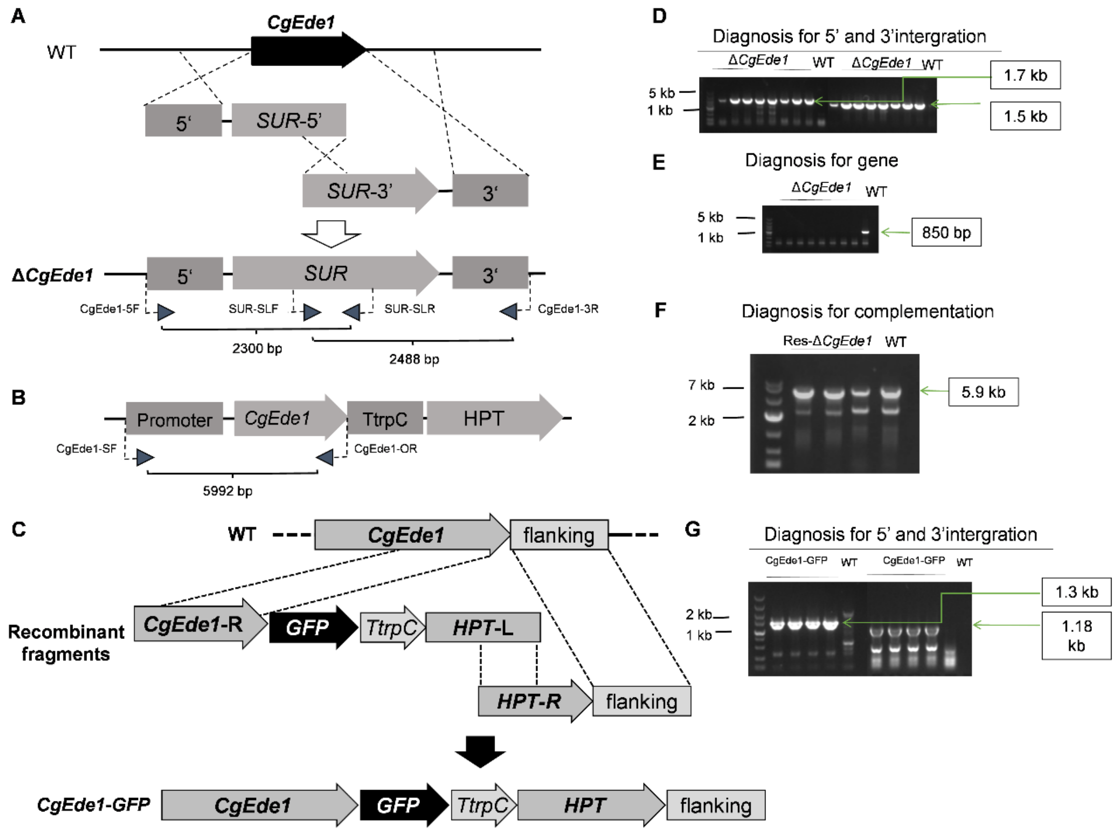

Figure S2 Construction of the *CgEde1* knock-out, complementary and GFP fusion strains. (**A**) Split-Marker strategy for the construction of *CgEde1* knock-out mutant. Diagnostic primers for integrations of the recombinant fragments are marked with black triangles. (**B**) Strategy for the construction of complementation strain Res-Δ*CgEde1*. (**C**) Strategy for the construction of CgEde1-GFP expressing strain. (**D**) Diagnosis for integrations of the recombinant fragments into the *CgEde1* gene locus. (**E**) Diagnosis for the detection of *CgEde1* nucleotide sequences in the knock-out mutants. (**F**) Diagnosis for the detection of complemented strain. (**G**) Diagnosis for the detection of CgEde1-GFP expressing strains. Δ*CgEde1*: the knock-out mutant; WT: wild type.

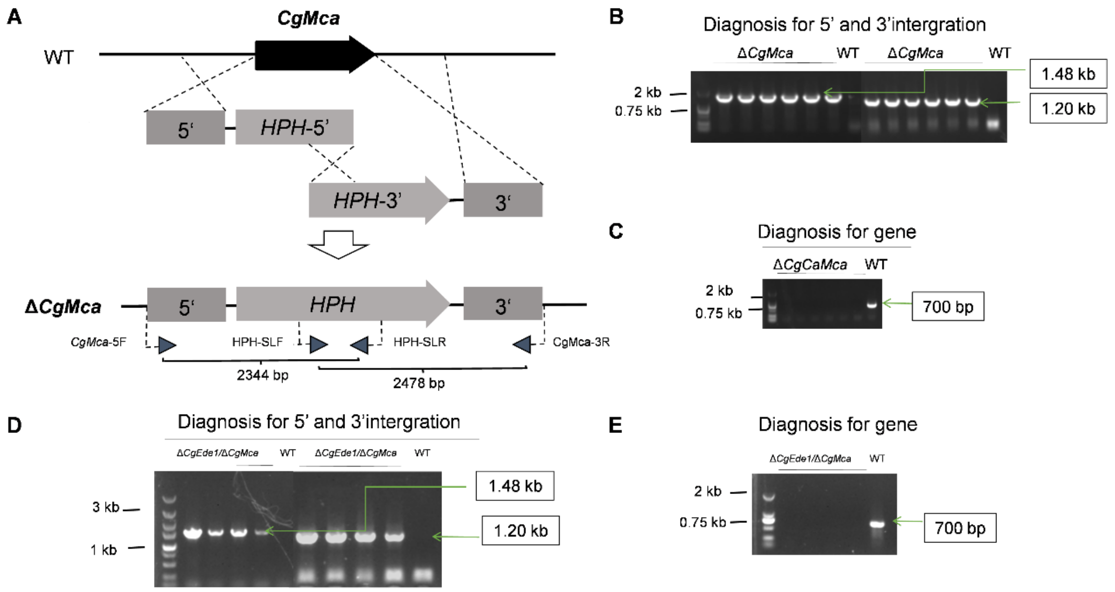

Figure S3 Strategies for the construction of Δ*CgMca* and double mutant Δ*CgEde1*/Δ*CgMca* strains. (**A**) Diagnosis for integrations of the recombinant fragments into the *CgMca* gene locus of Δ*CgMca*. (**B**) Diagnosis for the detection of *CgMca* nucleotide sequences in the knock-out Δ*CgMca* mutants. (**C**) Diagnosis for integrations of the recombinant fragments into the *CgMca* gene locus in double mutant Δ*CgEde1*/Δ*CgMca*. (**D**) Diagnosis for the detection of *CgMca* nucleotide sequences in double mutant Δ*CgEde1*/Δ*CgMca*.

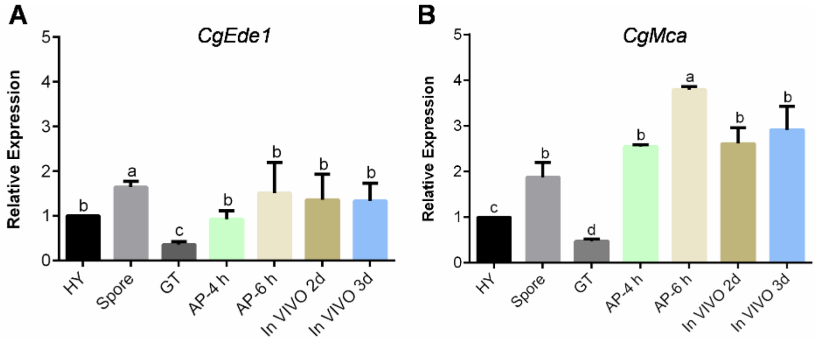

Figure S4 RT-qPCR analysis of *CgEde1* and *CgMca* expression patterns. (**A**) RT-qPCR analysis of *CgEde1* expression patterns. (**B)** RT-qPCR analysis of *CgMca* expression patterns. Gene expressions in Hypha (HY), Spore, geminated tube (GT), appressoria on cellophane (AP-4 h, AP-6 h), and inoculated leaves (*in vivo* 2d, 3d) using *β2-tublin* gene for normalization. Results are presented as average fold change of three technical repetition compared to the Hypha sample. Error bars represented SD. Asterisks represent significant differences (An unpaired one-tailed t test was performed. $P < 0.01$).

Table S1 Primers used in the study.

primer	sequence	application
CgEde1-5F	TTCGGATCTGCATTG	Knock out of *CgEde1*
CgEde1-MF1	GAGTTTCACGCCGCCATCGTGCCAACGCCACAGTG	
CgEde1-MR1	CACTGTGGCGTTGGCACGATGGCGGCGTGAAACTC	
CgEde1-MF2	GAATTGCATGCTCTCACACCTGATCTAGATGTGTT	
CgEde1-MR2	AACACATCTAGATCAGGTGTGAGAGCATGCAATTC	
CgEde1-3R	GGTCGGGTTAGACGATA	
sur-SLR	ATGTTGGCATAAGCCGAACCGT	
sur-SLF	CCTCTGATATTGGAAGCGACGC	
CgEde1-JC5F	AAGTCGCCCTCCATTCTTCTC	Diagnosis of mutant Δ*CgEde1*
SUR-JC5R	GCGTTTGTAACTCTGCCTGTTTG	
SUR-JC3F	ACGAGGACCGCTACTCACATAC	
CgEde1-JC3R	TCATTGTCAGGCAGACAGAGC	
CgEde1-OF	CAGAAAGGACTCGTCGCTAT	
CgEde1-OR	GCTCTGTCCCGTTGTTGTAG	
CgEde1-oF-XbaI	TCTAAGATTIGGATGCGGAAGAAG	Complementation strain construction of *CgEde1*
CgEde1-oR-BamHI	GGATCCTGATTGACTGGCAAGGAA	
HPH-SLF	CGTTGCAAGACCTGCCTGAA	
HPH-SLR	GGATGCCTCCGCTCGAAGTA	
CgMca-5F	CCACCAAACCGAATCCT	Knock out of *CgMca*
CgMca-MF1	AGGACCATATCTCCAGGATGAAAAAGCCTGAACT	
CgMcae-MR1	AGTTCAGGCTTTTTCATCCTGGAGATATGGTCCT	
CgMca-MF2	CCGACCGGGAACCAGTTTATACCCCACGTAACGG	
CgMca-MR2	CCGTTACGTGGGGTATAAACTGGTTCCCGGTCGG	
CgMca-3R	TTCACTACTACCCATCACC	
HPH-SPF	CGTTGCAAGACCTGCCTGAA	
HPH-SPR	GGATGCCTCCGCTCGAAGTA	
caspase-JC5F	CGGTGCAACACTGAATGGAAG	Diagnosis of mutant Δ*CgMca* Δ*CgEde1*/Δ*CgMca*
caspase-JC3R	ATGGTCCTGTGAAAGGTGTTGAG	
hphJC3F	CCGTGGTTGGCTTGTATGGAG	
hphJC5R	ACCCGCTCGTCTGGCTAAGAT	
caspase-OF	GCCTCAGCCCAATTACAACAAC	
caspase-OR	ATTCAAACATAGGAGCCAACCC	
Ede13'-HPH-MF	CCGACCGGGAACCAGTTACCTGATCTAGATGTGT	Construction of CgEde1-GFP expressing strain
Ede13'-HPH-MR	ACACATCTAGATCAGGTAACTGGTTCCCGGTCGG	
CgEde1-SF-XbaI	TCTAGACTCCCACGGTTGAAAGC	
CgEde1-oR-BamHI	GGATCCTGATTGACTGGCAAGGAA	
HPH-SPLF	CGTTGCAAGACCTGCCTGAA	
HPH-SPLR	GGATGCCTCCGCTCGAAGTA	
CgEde1-3R	GGTCGGGTTAGACGATA	

CgEde1-F	GCCGCAGTCCTCCTTTGATG	Expression of *CgEde1*
CgEde1-R	GCCCATGCTCGTGAGGTTCT	
CgMca-F	TTTCGATTCGTGTCACTCCG	Expression of *CgMca*
CgMca-R	CGATTGTGGGCATCTTCTCC	
CgHOX2-F	TGCCTGACGAACCTATACAC	Expression of conidiation-related genes
CgHOX2-R	TGAAGACGCATCTGACCAAT	
CgHOX4-F	GATAGCGGACGGAAGTTTGG	
CgHOX4-R	CCTTGTAGTGGCCCTTGAGC	
CgHOX7-F	CCACCCTAGCCGAGTCCAGT	
CgHOX7-R	ACGCACGCAGCTTGTCAGTC	
CgCon6-F	GGCTGGCGACAACGAGGACA	
CgCon6-R:	ACGCTCCTTGGCGGACTGCT	
CgCon-10-F	AACCGGCCCAAGGAGGAAGT	
CgCon10-R	AGCAGAACCGCTGGCGAGAC	
CgCon8-F	AACAGAAGCGTCACGATGGC	
CgCon8-R	CCACGAGTCCAGTTCTGCCA	
CgCOM1-F	CACCGCCTACGAATGCTTCA	
CgCOM1-R	GGTCCGCCATCTTGTCCTCA	
CgStuA-F	ATTCCAGTGGGCACAGGGTC	
CgStuA-R	CTGTTGGGCGGAAGGGTTAT	
CgCOS1-F	CAGCAACAAGGGTATCAAGG	
CgCOS1-R	GTCCAAGTCGGTGTCGTAAA	
